# Impact of *FHIT* loss on the translation of cancer-associated mRNAs

**DOI:** 10.1186/s12943-017-0749-x

**Published:** 2017-12-28

**Authors:** Daniel L. Kiss, William Baez, Kay Huebner, Ralf Bundschuh, Daniel R. Schoenberg

**Affiliations:** 1 Center for RNA Biology, The Ohio State University, Columbus, OH 43210 USA; 2 Comprehensive Cancer Center, The Ohio State University, Columbus, OH 43210 USA; 3 Department of Biological Chemistry and Pharmacology, The Ohio State University, Columbus, OH 43210 USA; 4 Department of Physics, The Ohio State University, Columbus, OH 43210 USA; 5 Department of Cancer Biology and Genetics, The Ohio State University, Columbus, OH 43210 USA; 6 Department of Chemistry & Biochemistry, The Ohio State University, Columbus, OH 43210 USA; 7 Division of Hematology, Department of Internal Medicine, The Ohio State University, Columbus, OH 43210 USA; 8 Biomarker Research Program, Houston Methodist Research Institute, Houston, TX 77030 USA

**Keywords:** Fhit, Translational control, Ribosome profiling, Scavenger decapping, Gene expression

## Abstract

**Background:**

*FHIT* is a genome caretaker/tumor suppressor that is silenced in >50% of cancers. Although it was identified more than 20 years ago, questions remain as to how *FHIT* loss contributes to cancer, and conversely, how *FHIT* acts to maintain genome integrity and suppress malignancy. Fhit belongs to the histidine triad family of enzymes that catalyze the degradation of nucleoside 5′,5′-triphosphates, including the m^7^GpppN ‘caps’ that are generated when mRNAs undergo 3′-5′ decay. This raised the possibility that Fhit loss might affect changes in the translation of cancer-associated mRNAs, possibly as a consequence of increased intracellular concentrations of these molecules.

**Results:**

Ribosome profiling identified several hundred mRNAs for which coding region ribosome occupancy changed as a function of Fhit expression. While many of these changes could be explained by changes in mRNA steady-state, a subset of these showed changes in translation efficiency as a function of Fhit expression. The onset of malignancy has been linked to changes in 5’-UTR ribosome occupancy and this analysis also identified ribosome binding to 5′-untranslated regions (UTRs) of a number of cancer-associated mRNAs. 5’-UTR ribosome occupancy of these mRNAs differed between Fhit-negative and Fhit-positive cells, and in some cases these differences correlated with differences in coding region ribosome occupancy.

**Conclusions:**

In summary, these findings show Fhit expression impacts the translation of a number of cancer associated genes, and they support the hypothesis that Fhit’s genome protective/tumor suppressor function is associated with post-transcriptional changes in expression of genes whose dysregulation contributes to malignancy.

**Electronic supplementary material:**

The online version of this article (10.1186/s12943-017-0749-x) contains supplementary material, which is available to authorized users.

## Background

‘Fragile’ sites are among the most frequently deleted loci in cancers [1]. *FHIT* was identified more than 20 years ago [2] at a locus that is deleted or otherwise silenced in >50% of most human cancer types [3, 4]. Loss of *FHIT* alleles occurs early in malignant transformation [5, 6], and is associated with decreased apoptosis [7], increased EMT [8, 9], increased resistance to genotoxic agents [10], and altered control of reactive oxygen species production [11]. Nevertheless, the mechanisms through which Fhit protein affects these functions have remained elusive.

Fhit is a small cytoplasmic protein that does not interact with known tumor suppressors or oncogenes. Its name (Fragile Histidine Triad) derives from a His-X-His-X-His-XX motif characteristic of nucleoside hydrolases. Fhit cleaves diadenosine triphosphate (Ap3A) in vitro to yield ADP and AMP [12], and Ap3A accumulates in Fhit-deficient cells [13]. Recently Taverniti and Seraphin [14] identified Fhit as a scavenger decapping enzyme. Scavenger decapping enzymes are responsible for degrading m^7^GpppN cap dinucleotides that are generated by 3′-5’ mRNA decay. Because they can be bound by eIF4E such mRNA decay remnants can inhibit translation initiation if they accumulate to a level that can compete with mRNA 5′ ends. DcpS is the major scavenger decapping enzyme [15], and the identification of Fhit as another of this type of enzyme is consistent with previous work describing Fhit’s ability to cleave GpppBODIPY, an mRNA cap-like molecule [16].

Translation plays a critical role in cancer [17], and a recent report showed that, for a number of cancer-associated mRNAs, changes in ribosome occupancy of upstream open reading frames precedes the appearance of detectable tumors [18]. As noted above, the key biochemical property of Fhit is its ability to hydrolyze nucleoside 5′,5′-triphosphates, and while catalytically-inactive forms of Fhit are unable to function as tumor suppressors, a mutant (H96N) that binds to but does not hydrolyze nucleoside 5′,5′-triphosphates is nearly as effective as wild-type Fhit in suppressing tumor formation [12]. *FHIT* allele loss can occur prior to appearance of detectable preneoplastic lesions or tumors [5, 6, 19]. Since few direct targets of Fhit loss have been identified [11, 20], we wondered if its tumor suppressor or genome caretaker effects might be related to its function in degrading nucleoside 5′,5′-triphosphates, and to the impact of these molecules on the translation of a limited number of mRNAs.

The current study used Fhit expression-negative H1299 lung cancer cells carrying an inducible *FHIT* transgene to examine the impact of Fhit protein and its loss on the scope of translating mRNAs. Ribosome profiling and RNA-Seq showed that Fhit loss is associated with changes in steady-state level and ribosome occupancy of several hundred mRNAs, but little change in translation efficiency of most of the transcriptome. Rescuing Fhit expression resulted in changes in average ribosome density of a limited number of cancer-associated genes and this was reflected by changes in protein expression. We also identified additional cancer-associated genes for which 5’-UTR ribosome occupancy changed when Fhit expression was restored. This result is consistent with transformation-associated changes in 5’-UTR ribosome occupancy in [18] and with loss of Fhit expression as an early step in this process.

## Methods

### Ribosome profiling, RNA-seq, and informatics

Detailed description of the methods used for ribosome profiling and data analysis are in Additional file 1.

### Cell culture

The engineered H1299 lung carcinoma cells, which inducibly express Fhit cDNA and protein (D1) or an empty vector (E1) have been described [20]. H1299 cells were maintained in DMEM medium with 10% FBS, zeocin, gentamicin and geneticin. Fhit expression was induced by addition of ponasterone A (2 μM) (Life Technologies) to the growth medium for 48 h.

### Preparation of cell extracts and RNA

Cytoplasmic extracts were prepared and harvested as described in [21]. Briefly, H1299 E1 and D1 cells (1.75 × 10^6^ cells) were seeded into 150 mm dishes. 24 h later, the cells were induced with ponasterone A (2 μM) for 48 h. Cells were washed twice with PBS, scraped, and pelleted by centrifugation at 2500 xg for 5 min at 4 °C. Subsequent procedures were carried out on ice. Cell pellets were lysed in 5 pellet volumes of ice cold cytoplasmic lysis buffer (50 mM Tris-HCl, pH 7.5, 10 mM KCl, 10 mM MgCl_2_, 150 mM NaCl, 0.2% NP-40, 2 mM dithiothreitol (DTT), 0.5 mM phenylmethylsulfonyl fluoride (PMSF), 1 mM sodium orthovanadate, supplemented with 5 μl/ml RNAseOUT (Life Technologies), 25 μl/ml protease inhibitor cocktail (Sigma), 10 μl/ml each phosphatase inhibitor cocktails 2 and 3 (Sigma)). Lysates were incubated on ice for 10 min with mild agitation every 2 min. Nuclei and other debris were pelleted with a 10 min centrifugation at 4 °C and 16,100 xg. Pellets were discarded and supernatants were reserved and used for western blotting (see below) or RNA extraction. Cytoplasmic RNA was harvested from the supernatants using a Direct-Zol (Zymo) kit according to the manufacturer’s Reaction Clean-Up protocol. Whole cell extracts were prepared as above, but nuclei were disrupted with three 10 s pulses (5 min rest on ice between each pulse) using a Fisher Scientific 60 sonic dismembrator fitted with a microtip on setting 3 prior to the final centrifugation step.

### Western blotting

SDS Laemmli loading dye was added to cytoplasmic or whole cell extracts harvested as above and samples were heated at 95 °C for 5 min. Proteins were separated by SDS gel electrophoresis, transferred to Immobilon FL (EMD millipore) PVDF membranes, and immunoblotted with antisera against human Fhit (1:1000, [22], human eEEF2 (1:1000, One World Labs), human TP53I3 (1:1000, One World Labs), human IFIT1 (1:600, Protein-Tech), human Vinculin (1: 5000, Abcam), human ADAM9 (1:1000, GeneTex) and human FASN (1:1000, Proteintech). IR-680 or IR-800 conjugated goat anti-rabbit or anti-mouse secondary antibodies were used at 1:5000 dilutions and blots were scanned and bands analyzed using a Licor Odyssey Imager.

### Reverse transcription and quantitative PCR (qPCR)

For each sample, one microgram of cytoplasmic RNA was spiked with 0.1 ng of Luciferase control RNA (Promega), and reverse transcribed using Superscript III (Life Technologies) with random hexamers according to the manufacturer’s instructions. cDNA was assayed using a CFX Connect Real-Time System (BioRad) for qPCR. Briefly, the cDNA from RT-PCR was diluted 10-fold and qPCR reactions were carried out using SensiFAST SYBR No-ROX reagent (Bioline) following the manufacturer’s protocol for a 2-step reaction. Predesigned Primetime qPCR primer pairs for GAPDH (Hs.PT.39a.22214836), eEF2 (Hs.PT.58.40025410), TP53i3 (Hs.PT.58.27248188), IFIT1 (Hs.PT.56a.20769090.g) and custom primer pairs for Luciferase and Vinculin were purchased from Integrated DNA Technologies (IDT). Primer sequences and ordering information are listed in Additional file 2. Primer efficiencies were determined using a standard curve from serial dilutions and efficiency was calculated using Efficiency = −1 + 10^(−1/slope)^. The comparative ΔΔCt method (2^-ΔΔCq^) was used to calculate the relative mRNA levels (normalized against the spiked luciferase and an endogenous control (GAPDH) transcript) in empty vector (E1) and Fhit-expressing (D1) cells. All qPCR assays were performed in triplicate using cDNA reverse transcribed from independent biological triplicate RNA samples.

### uORF predictions

The sequences of the entire 5’-UTR and coding region were analyzed using the **seqshoworfs** command within MATLAB’s (Ver: 8.1.0.60) Bioinformatics Toolbox (Ver: 4.3), with the “AlternativeStartCodon” search parameter set to true, to look for start codons: ATG, GTG, CTG, TTG; reading frames: 1, 2, and 3 on the direct strand only; and searched for uORFs with a minimum length of 10 codons using the standard genetic code.

## Results

### Ribosome profiling identifies a set of mRNAs whose translation is controlled by Fhit

**Fig. 1:**
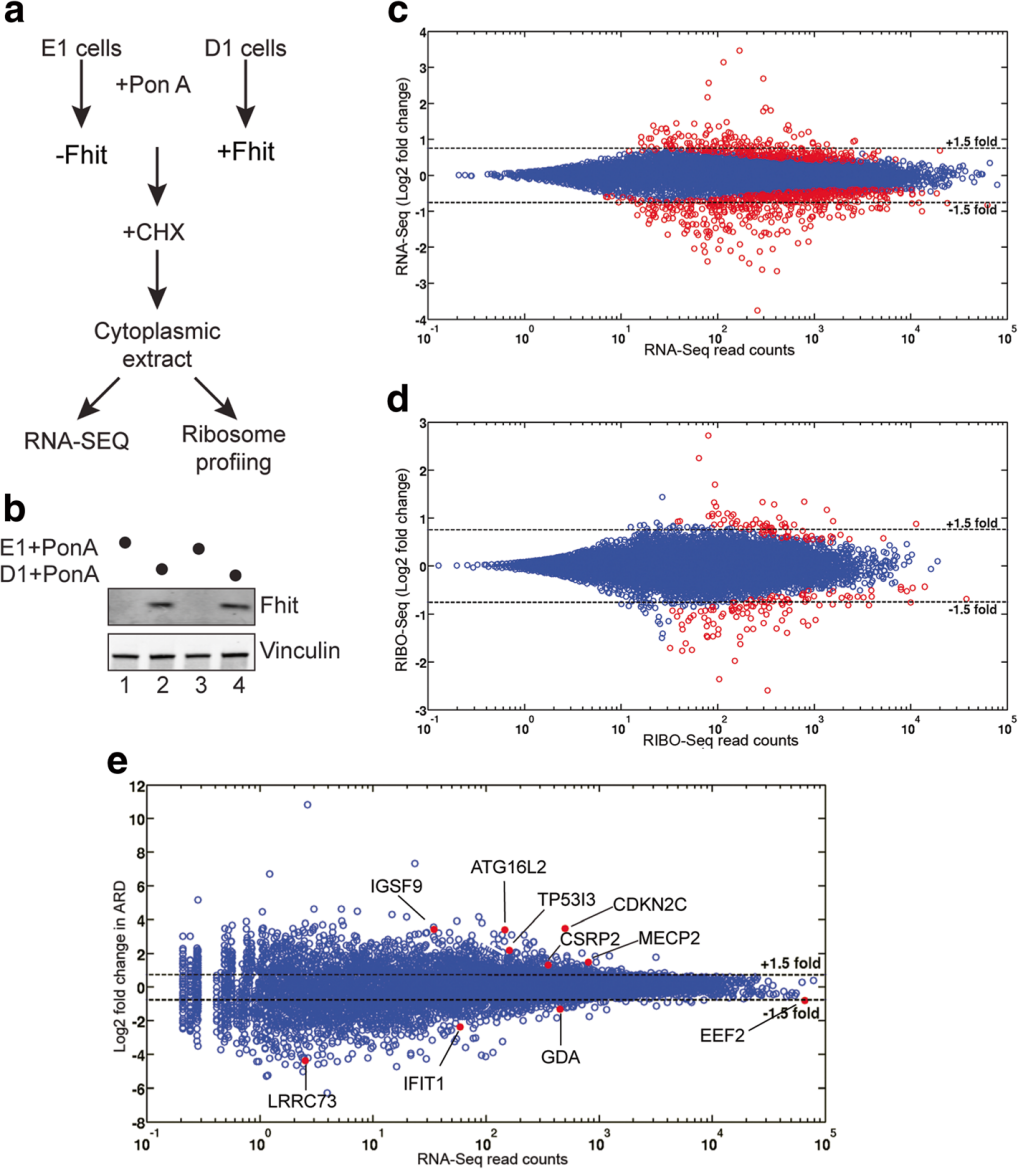
Identification of mRNAs whose levels and translation are altered by Fhit**. a** The workflow for identifying mRNAs under translational control by Fhit is shown. E1 cells are H1299 cells with a stably transfected empty vector and D1 cells are H1299 cells with a stably transfected, ponasterone A-inducible *FHIT* transgene. Duplicate cultures of each cell line were treated with Ponasterone A, and cytoplasmic extracts were used for RNA-Seq and ribosome profiling (RIBO-Seq). **b** Fhit induction in D1 but not E1 cells was confirmed by Western blotting. **c** The log_2_ change in steady state levels of the mRNA transcriptome is shown as a function of RNA-Seq read counts. All mRNAs that underwent a statistically-significant change in association with Fhit expression (*p* < 0.05) are indicated with red circles, and we arbitrarily selected a 1.5-fold change as a cutoff for further study. These transcripts and their fold changes are listed in Additional file 4. **d** The log_2_ fold change in coding region RIBO-Seq for each mRNA is shown as a function of RNA-Seq read counts. Statistically significant changes are indicated by red circles as in **c**. These transcripts and their fold changes are listed in Additional file 7. **e** RiboDiff was used to normalize the coding region RIBO-Seq data in **d** to the RNA-Seq data in **c**. Fhit-mediated changes in the resulting Average Ribosome Density (ARD) are plotted as a function of RNA-Seq read counts. Again a cutoff of 1.5-fold was selected. mRNAs that underwent a statistically-significant change with Fhit (*p* < 0.05) are labeled and indicated with red dots

The approach used to determine the transcriptome-wide impact of Fhit expression on translation is shown in Fig. 1a. The E1 cells are Fhit-negative H1299 cells stably transfected with empty vector (E1 cells), and the D1 cells carry a Ponasterone A-inducible *FHIT* transgene. Prior to generating sequencing libraries we confirmed that treatment with Ponasterone A induced Fhit expression in the D1 cell line but not the E1 cell line (Fig. 1b). Duplicate cultures of each line were then processed for RNA-Seq and ribosome profiling (RIBO-Seq) [23], with all data subjected to Benjamini Hochberg False Discovery Rate correction. Scatter plots showed good agreement between duplicate RNA-Seq libraries of E1 and D1 cell lines (Additional file 3). Fhit expression had limited overall impact on the transcriptome, with most mRNAs showing no statistically significant change in their steady-state level (Fig. 1c). Using a 1.5-fold change as cutoff (red circles) 209 transcripts increased and 377 decreased significantly in Fhit-positive vs Fhit-negative cells. The identities and changes in the abundance of these mRNAs are listed in Additional file 4.

Scatter plots of RIBO-Seq data also showed good agreement between duplicate D1 and E1 RIBO-Seq libraries (Additional file 5). The quality of these libraries was further supported by a metagene analysis of ribosome protected fragments that showed the expected triplet phasing near the start and stop codons and a peak 13 nucleotides upstream of start codons characteristic of ribosomes bound to this site (Additional file 6). Fhit expression also significantly impacted the quantity of ribosome-bound transcripts (Fig. 1d), with 67 transcripts showing increased and 103 transcripts showing decreased ribosome occupancy with Fhit expression. The identities and changes in ribosome occupancy of these mRNAs are listed in Additional file 7.

The FHIT-induced changes observed by RIBO-Seq could result from changes in translation efficiency, steady-state mRNA levels, or a combination of those factors. To decipher this, the RIBO-Seq data for the coding regions were normalized to RNA-Seq data using RiboDiff [24]. The resulting Average Ribosome Density (ARD), accurately tracks translation efficiency of each mRNA [25]. Scatter plots showed good agreement between duplicates of Fhit-positive (D1) and Fhit-negative (E1) cells (Additional file 8), which were then used to compare coding region ARD between treatment groups (Fig. 1e). This analysis identified 6 mRNAs with a statistically-significant increased coding region ARD in Fhit-positive vs Fhit-negative cells, and 4 mRNAs with decreased coding region ARD. These mRNAs and the degrees to which coding region ARD changed with Fhit are presented in the top half of Table 1.

**Table 1:** mRNAs showing Fhit-dependent changes in ribosome loading

Symbol	Gene ID	Log_2_ D1/E1
Coding region		
CDKN2C	NM_001262	3.47
IGSF9	NM_020789	3.42
ATG16L2	NM_033388	3.39
TP53I3	NM_004881	2.18
MECP2	NM_004992	1.48
CSRP2	NM_001321	1.31
EEF2	NM_001961	−0.81
GDA	NM_001242505	−1.31
IFIT1	NM_001548	−2.37
LRRC73	NM_001012974	−4.39
5’-UTR		
ACSL1	NM_001995	3.69
TROVE2	NM_004600	3.47
ADAM9	NM_003816	1.51
HIST1H2AD	NM_021065	1.30
H2AFZ	NM_002106	0.72
RACK1	NM_006098	−0.88
RPL37A	NM_000998	−2.01

The Average Ribosome Density (ARD) for Ponasterone A-treated E1 and D1 cells was determined using RiboDiff and only those transcripts with an adjusted *p* value <0.05 were considered further. Column 3 shows the log_2_ induction ratio + Fhit(D1)/ -Fhit (E1). The upper panel lists mRNAs with changes in ribosomes bound to coding regions. The lower panel lists mRNAs with changes in ribosomes bound to the 5’-UTR using a cutoff of 25 nucleotides upstream of the annotated start codon

### Association of *FHIT* loss with changes in coding region ribosome occupancy

**Fig. 2:**
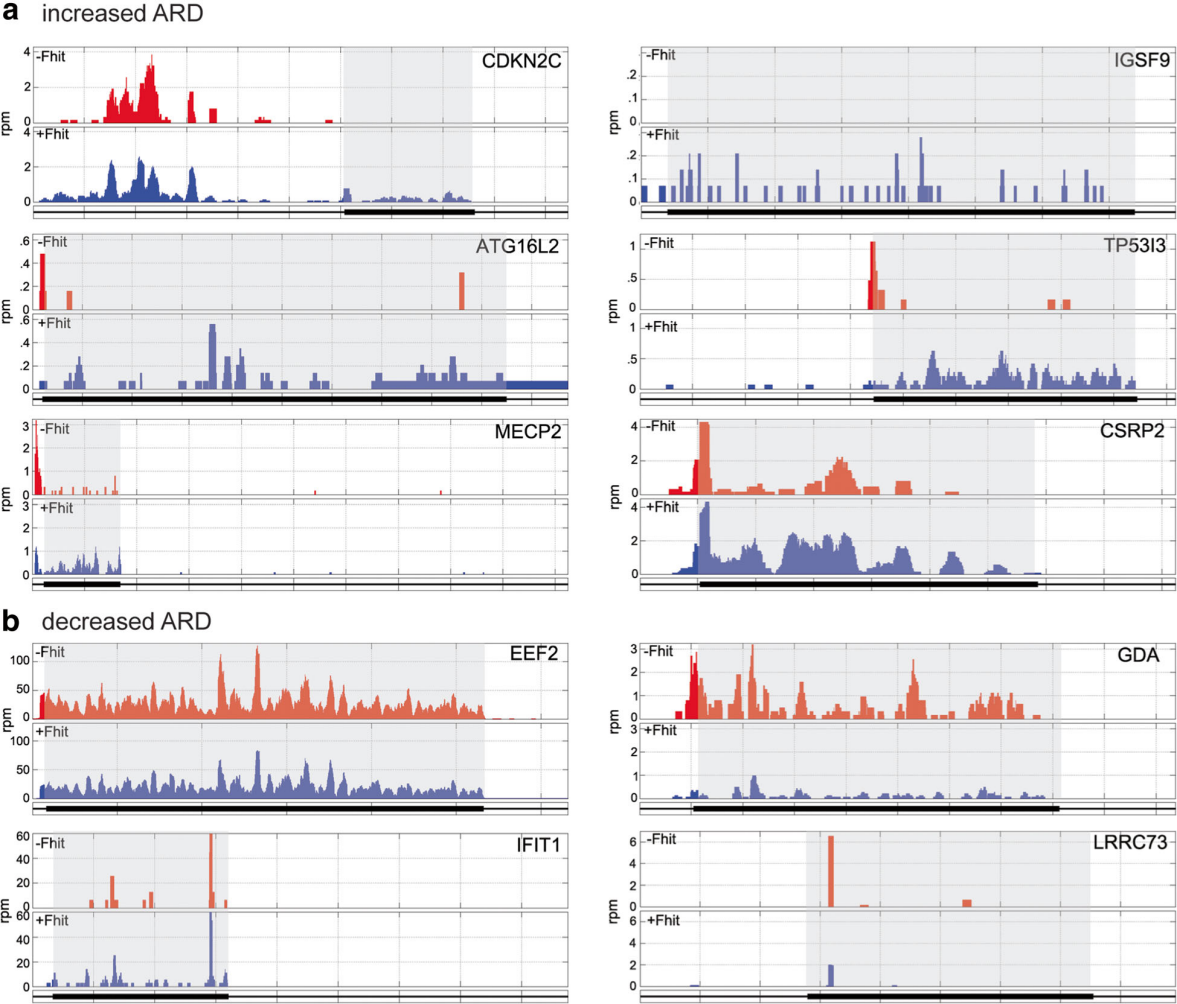
Fhit-mediated changes in ribosome distribution across mRNAs identified by coding region ARD**. a** Ribosome distribution is shown across the mRNAs identified in Table 1 as having increased coding region ARD in Fhit-positive (blue) vs Fhit-negative (red) cells. **b** Ribosome distribution is shown across the mRNAs identified in Table 1 as having decreased coding region ARD in Fhit-positive (blue) vs Fhit-negative (red) cells. The coding region of each mRNA is shaded

Plotting ribosome distribution across each of these mRNAs identified positional changes in ribosome occupancy as one mechanism by which Fhit loss/Fhit expression affects ARD (Fig. 2). CDKN2C mRNA has a short (506 nt) coding region and a long (1216 nt) 5’-UTR that includes several putative upstream open reading frames (uORFs). In Fhit negative cells CDKN2C showed little evidence for CDS ribosome occupancy; however, ribosomes were present on the 5’-UTR. 5’-UTR ribosome occupancy decreased with Fhit expression and this was accompanied by the appearance of CDS-bound ribosomes, suggesting one or more features of the CDKN2C 5’-UTR are responsible for the Fhit-mediated increase in translation (see below). Similar results were also observed for ATG16L2 and MECP2. In Fhit-negative cells there was little evidence for translating ribosomes on the CDS of IGSF9 and TP53I3, and for both of these mRNAs Fhit expression resulted in the appearance of bound ribosomes. TP53I3 was unique in having stalled ribosomes at the initiation codon that disappear and are replaced by ribosomes that distribute across the entire coding region in Fhit-expressing cells. Taken together, these data support a role for Fhit in maintaining the translation of a limited set of mRNAs.

RiboDiff analysis also identified a number of mRNAs whose ARD was elevated in Fhit-negative versus Fhit-positive cells, the most abundant of which was EEF2 (Fig. 2b). Importantly, EEF2 is elevated in lung squamous cell carcinoma and elevated levels of EEF2 drive cell proliferation and promote EMT [26].

### Validation of ARD targets

**Fig. 3:**
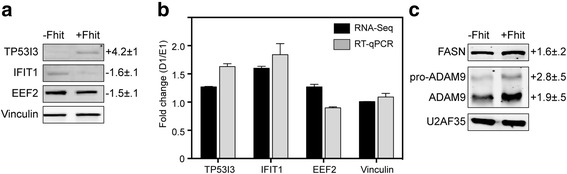
Fhit-mediated changes in target protein expression are independent of mRNA level. **a** D1 and E1 cells treated for 24 h with Ponasterone A were analyzed for changes in TP53I3, IFIT1, EEF2 and Vinculin. Shown are representative Western blots for each of triplicate determinations and the fold change normalized to Vinculin is presented beneath the panels for TP53I3, IFIT1 and EEF2. **b** Cytoplasmic extracts from these cells were spiked with luciferase RNA as a control for sample recovery and analyzed by RT-qPCR for changes in steady-state levels of TP53I3, EEF2, IFIT1 and Vinculin mRNA. The mean fold change ± SEM (*n* = 3) determined for each mRNA by by RT-qPCR (black bars) is shown alongside the average difference from the duplicate RNA-Seq datasets. **c** The analysis in **a** was repeated for ADAM9 and FASN, with U2AF35 used as a normalization control

To confirm that the preceding results reflect changes in protein expression 3 of the targets in Table 1 were selected (on the basis of antibody availability) for evaluation by Western blotting and RT-qPCR. Western blotting showing TP53I3 was 4.2-fold higher in Fhit-positive vs Fhit-negative cells. This was similar to the 4.5-fold difference in ARD determined by RiboDiff (Fig. 3a). In Fhit-expressing cells EEF2 was 1.5-fold lower by Western blotting and 1.7-fold lower by RiboDiff. In contrast, IFIT1 was 1.6-fold lower by Western blotting and 5.2-fold lower by RiboDiff. The reason for this difference is not known. Vinculin expression was unchanged in both RNA-Seq and RIBO-Seq (Additional files 4 and 7), and quantitative changes for each of these proteins were determined by normalizing to Vinculin.

We also looked for Fhit-mediated differences in the corresponding mRNAs by RT-qPCR and compared these results with those obtained by RNA-Seq (Fig. 3b). Although TP53I3 mRNA was somewhat higher in Fhit-positive versus Fhit-negative cells (~1.5-fold higher by RT-qPCR and ~1.2-fold higher by RNA-Seq) this was significantly less than the increase in TP53I3 protein determined by Western blotting and ARD, and by the changes in ribosome distribution seen in Fig. 2a. Although IFIT protein was lower in Fhit-positive cells IFIT1 mRNA was higher (1.8-fold higher by RT-qPCR, 1.6-fold higher by RNA-Seq), and EEF2 protein was ~1.5 lower but EEF2 mRNA was either unchanged by RNA-Seq or slightly lower by RT-qPCR. Taken together the preceding data confirm that Fhit affects the expression of a limited number of proteins through changes in the translation of their corresponding mRNAs.

### Impact of Fhit expression on 5’-UTR ribosome occupancy

**Fig. 4:**
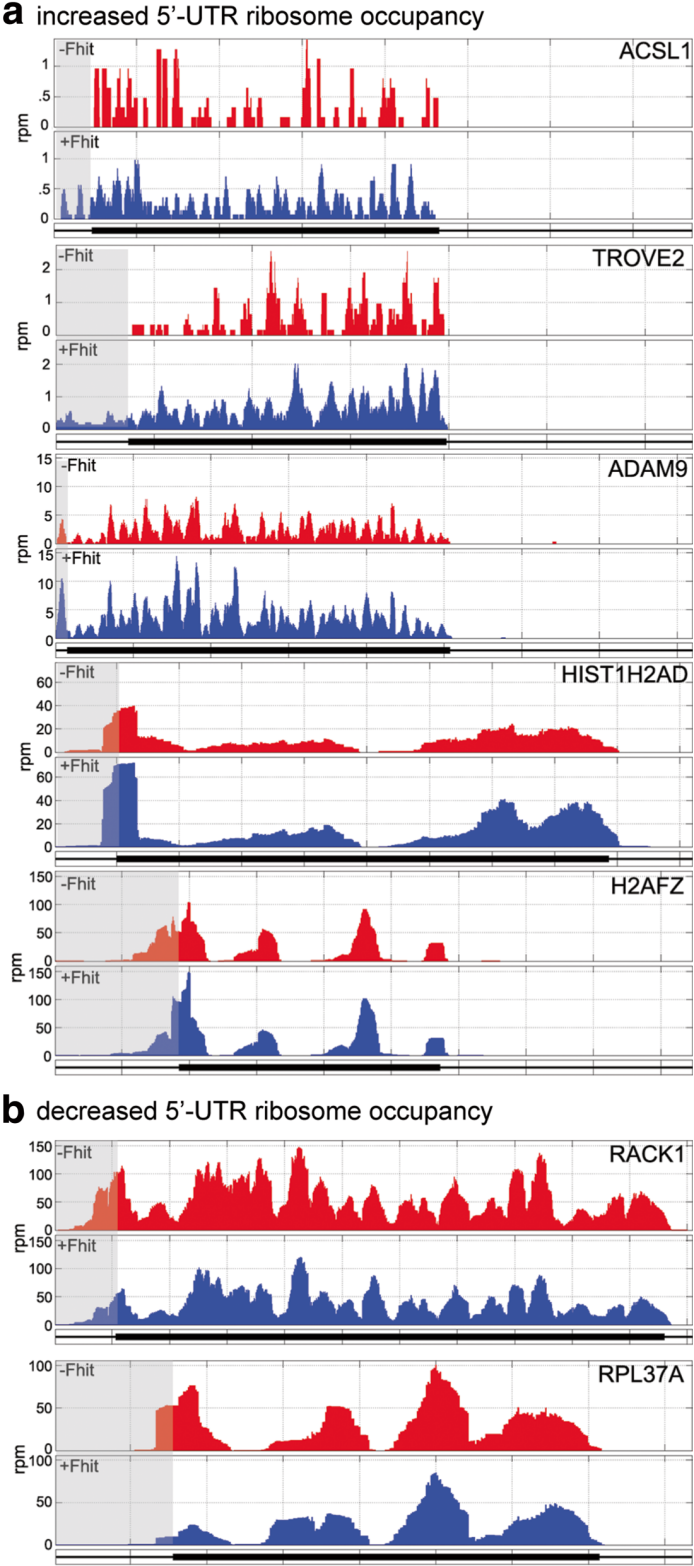
Fhit-mediated changes in ribosome distribution across mRNAs identified by changes in 5’-UTR ARD. **a** Ribosome distribution is shown across the mRNAs identified as having increased 5’-UTR ARD in Fhit-positive (blue) vs Fhit-negative (red) cells. **b** Ribosome distribution is shown across the mRNAs identified as having decreased 5’-UTR ARD in Fhit-positive (blue) vs Fhit-negative (red) cells. The 5’-UTR of each mRNA is shaded

The identification of 5’-UTR bound ribosomes on Fhit-regulated mRNAs was unanticipated and suggested a possible link between Fhit-mediated changes in gene expression and cancer-associated changes in 5’-UTR ribosome binding [18]. RiboDiff analysis of 5’-UTRs identified 5 mRNAs with increased 5’-UTR ribosome occupancy associated with Fhit expression, and 2 mRNAs with decreased 5’-UTR ribosome occupancy (bottom half of Table 1, Fig. 4). Importantly, each of these has one or more predicted uORFs within its 5’-UTR (Additional file 9). These changes ranged from a 13.6-fold increase for ACSL1 to a 4-fold decrease for RPL37A. In some cases differences in 5’-UTR ribosome occupancy were also associated with differences in CDS ribosome occupancy, an example of which is ADAM9. ADAM9 encodes a precursor that is processed to the mature protein, and Western blotting confirmed both forms were higher in Fhit-expressing cells (Fig. 3c).

### Fhit expression affects the relative representation of 5’-UTR vs coding region-bound ribosomes

**Fig. 5:**
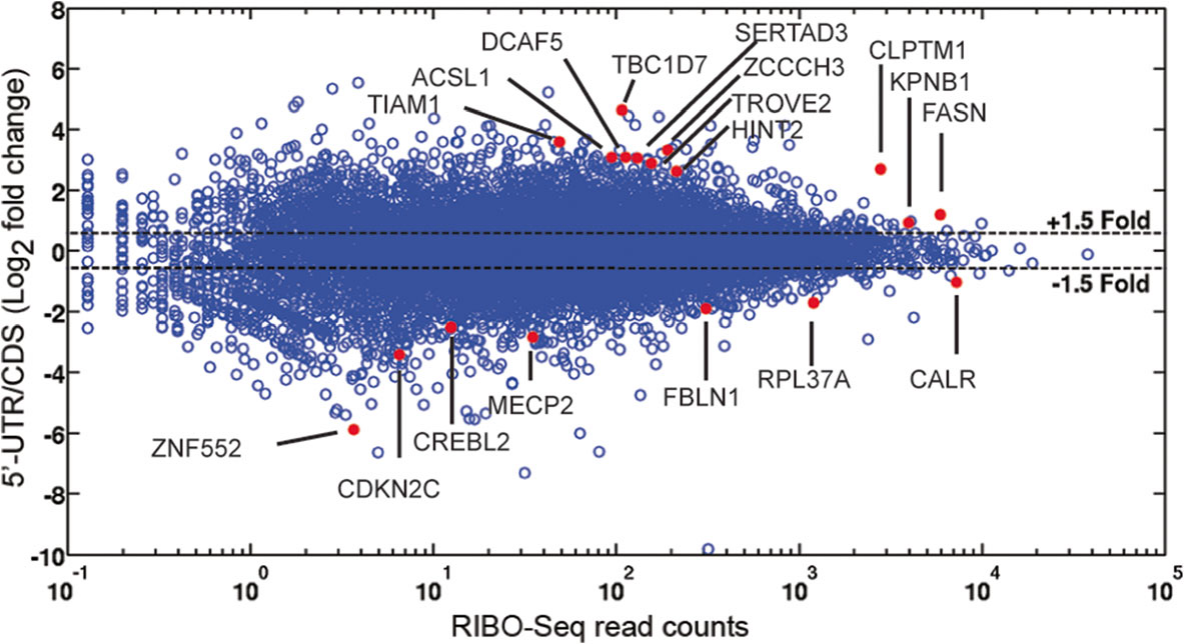
Identification of mRNAs with Fhit-mediated changes in ribosome occupancy of 5’-UTR versus coding region**.** RiboDiff was used to determine ribosome occupancy of 5’-UTRs vs the coding region. The results of which were used in Additional file 11 to identify mRNAs for which Fhit expression altered the ratio between these features. These data were plotted as a function of RIBO-Seq read counts, with those mRNAs from Additional file 11 showing statistically-significant changes identified by name and as red dots

**Fig. 6:**
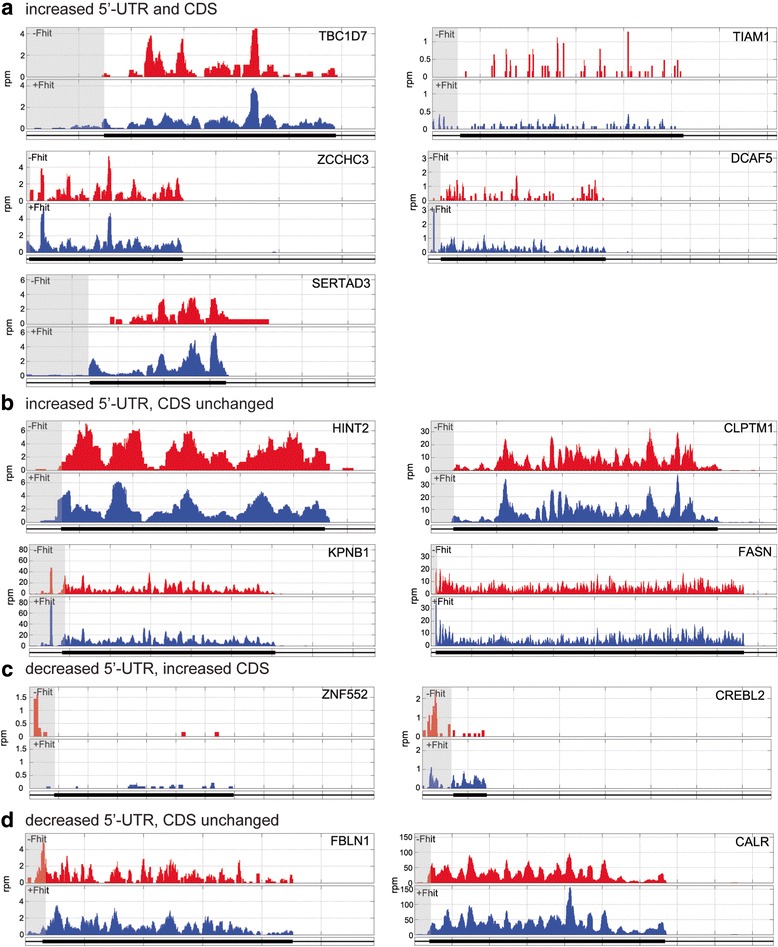
Fhit changes the ribosome occupancy ratio of 5’-UTR versus coding region. Ribosome distribution is shown across the mRNAs identified in Table 2 as having changes to 5’-UTR/coding region ribosome occupancy in Fhit-positive (blue) vs Fhit-negative (red) cells. Omitted are mRNAs from Table 2 whose ribosome distribution is presented in Fig. 4. The 5’-UTR of each mRNA is shaded. **a** Five mRNAs showed increased ribosome loading in both the 5’-UTR and the coding region, but the increases observed in the 5’-UTR were larger than those observed in the coding region. **b** Four mRNAs exhibited an increase in 5’-UTR-bound ribosomes while their coding regions remained essentially unchanged. **c** Two mRNAs showed decrease 5’-UTR ribosome occupancy in Fhit-positive versus Fhit-negative cells, but increased coding region ribosome occupancy. **d** Two mRNAs showed decreased 5’-UTR ribosome occupancy in Fhit-positive versus Fhit-negative cells but little change in coding region ribosome occupancy

Several of the genes that were identified in Fig. 1e also showed reciprocal differences in 5’-UTR ribosome binding. To determine the extent of this effect RiboDiff data were mined for changes in the ratio of 5’-UTR-bound ribosomes vs CDS-bound ribosomes (Additional file 10). This identified 19 genes (*p* < 0.05) for which the ratio of 5’-UTR/CDS ribosome occupancy either increased or decreased as a function of Fhit expression (Table 2 and Fig. 5). Several of these, including CDKN2C, ACSL1, TROVE2, MECP2 and RPL37A were identified earlier (see Figs. 2 and 4). The remaining genes fall into four groups; mRNAs whose 5’-UTR and CDS occupancy increased in Fhit-positive cells (Fig. 6a), mRNAs showing increased 5’-UTR but little change in CDS ribosome occupancy (Fig. 6b), and mRNAs with decreased 5’-UTR ribosome occupancy (Fig. 6c and d). Although FASN coding region ribosome occupancy was unchanged the level of the encoded protein was slightly higher in Fhit-positive cells (Fig. 3c).

**Table 2:** mRNAs showing Fhit-dependent changes in ribosomes bound to the 5’-UTR versus coding sequence

Symbol	Gene ID	Ratio of 5’-UTR/CDS (Log_2_ fold change)
TBC1D7	NM_016495	4.65
TIAM1	NM_003253	3.61
ZCCHC3	NM_033089	3.33
DCAF5	NM_003861	3.09
ACSL1	NM_001995	3.09
SERTAD3	NM_013368	3.06
TROVE2	NM_004600	2.89
CLPTM1	NM_001294	2.70
HINT2	NM_032593	2.63
FASN	NM_004104	1.19
KPNB1	NM_002265	0.92
CALR	NM_004343	−1.05
RPL37A	NM_000998	−1.71
FBLN1	NM_006486	−1.92
CREBL2	NM_001310	−2.51
MECP2	NM_004992	−2.82
CDKN2C	NM_001262	−3.41
ZNF552	NM_024762	−5.89

RiboDiff was used to determine relative changes in ribosome occupancy of 5’-UTR versus coding region as a function of Fhit expression, using ARD data from Ponasterone A-treated E1 and D1 cells. Only those transcripts with an adjusted *p* value <0.05 were considered further. The complete list of genes, their relative changes and statistical analysis are included in Additional file 10

## Discussion

Despite more than 1100 papers on Fhit the molecular mechanism(s) by which it acts to affect these processes are poorly understood. We approached this from the perspective that Fhit might act indirectly to alter gene expression as a function of its ability to clear nucleoside 5′,5′-triphosphates and act as a scavenger decapping enzyme [14]. We reasoned that Fhit loss might increase the intracellular levels of these dinucleotides, including the m^7^G caps generated by mRNA 3′-5′ decay, and these in turn might affect the translation of some mRNAs. To address this we performed ribosome profiling of Fhit-deficient H1299 lung cancer cells carrying an inducible *FHIT* transgene and a matching cell line carrying empty vector. Changes in Fhit expression impacted ribosome occupancy, with 67 mRNAs showing statistically-significantly increased ribosome binding and 103 mRNAs showing decreased binding (Fig. 1d and Additional file 7). For the most part changes in ribosome occupancy could be accounted for by corresponding changes in mRNA level. Once this was taken into account 6 mRNAs were identified for which Fhit increased coding region average ribosome density (ARD) and 4 mRNAs for which ARD decreased (Fig. 1e, Table 1).

This small number of mRNAs was unexpected, but their identity was significant. Of the mRNAs showing increased translation in association with Fhit expression, CDKN2C is a member of the INK4 cyclin-dependent kinase inhibitors with medium to low expression in lung cancers (see [27, 28]. IGSF9 is involved in cellular adhesion, TP53I3 is induced by TP53 and thought to generate reactive oxygen molecules, MECP2 binds methyl-CpG sites in chromatin and CSRP2 is a LIM domain protein thought to be involved in regulating cell growth. Mecp2 is also the key protein that is lost or mutated in Rett syndrome [29]. We also identified a number of mRNAs for which coding region ribosome occupancy was higher in Fhit-negative versus Fhit-positive cells. The greatest differential was seen for EEF2, a GTP-binding protein that catalyzes the movment of peptide-bound tRNAs from the ribosome A site to the P site during translation. This is notable because EEF2 is highly expressed in numerous cancers [30], where it promotes cell proliferation and EMT [26].

Because we were interested in the relationship between Fhit loss and protein expression our initial work focused on coding region ribosome occupancy. However, this changed with the report by Sendoel et al. [18], showing the onset of malignancy is preceded by changes in 5’-UTR ribosome occupancy of a number of cancer-associated mRNAs. This was first evident in Fig. 2, where CDKN2C, ATG16L2 and MECP2 had higher 5’-UTR ribosome occupancy in Fhit-negative vs Fhit-positive cells. Restoring Fhit expression shifted this pattern, resulting in higher coding region and lower 5’-UTR ribosome occupancy of these mRNAs. A related picture was seen for TP53I3, but in Fhit-negative cells there appears to be a single stalled ribosome bound at the start codon that is released by Fhit expression. These findings are consistent with results in [31] and [32] in which a uORF regulates downstream translation, which in our case is affected by the presence or absence of Fhit.

Futher examination identified 5 mRNAs for which changes in 5’-UTR ribosome occupancy increased with Fhit rescue, 2 mRNAs for which 5’-UTR ribosome occupancy decreased (Fig. 4), and 13 mRNAs for which Fhit expression changed the ratio of 5’-UTR vs coding region ribosome occupancy. Each of these mRNAs has a potential or verified uORF, the sequences of which are listed in Additional file 9. As in [18], these uORFs have mostly non-canonical (CUG, GUG, UUG) start codons, and some extend into the coding region, raising the possibility that loss of Fhit leads to changes in ribosome occupancy that can affect the protein products translated from these mRNAs.

In total, this study identified 30 genes for which *FHIT* loss alters ribosome occupancy of the 5’-UTR or the coding region of their respective mRNAs (Table 3). These are notable for the breadth of pathways they impact. A number of genes have been directly linked to malignancy, including ADAM9, CDKN2C, DCAF5, KPNB1, MECP2, RACK1, SERTAD3, TBC1D7, TP53I3 and TIAM1. H2AF7 was recently identified as a major regulator of EMT [33]. Others that have been indirectly linked to cancer include HIST1H2AD, two zinc finger proteins (ZCCHC3, ZNF552), and CSRP2, CREBL2, and SERTAD3. Intermediary metabolism and protein synthesis have essential roles in malignancy, and these processes are represented in our dataset by ACSL1 and FASN, which encode enzymes that function in fatty acid synthesis and lipid metabolism, and TROVE2, EEF2, RACK1 and RPL37A, each of which impact RNA metabolism or translation. This analysis also identified HINT2, which is a histidine triad containing protein that functions in mitochondrial cell death signaling. Based on results presented here we propose that past challenges in identifying how loss of *FHIT* leads to cancer may be partially due to the fact that Fhit and its loss exert pleiotropic effects on the translation of genes that function in a multitude of cellular processes.

**Table 3:** Targets of Fhit-mediated changes in ribosome occupancy

Gene	Function	Changes in ribosome occupancy with Fhit induction
ACSL1	Acyl-CoA synthetase, functions in lipid metabolism by converting long chain fatty acids into their CoA esters	Increased 5’-UTR
ATG16L2	Autophagy Related 16 Like 2, paralog of ATG16L1, which functions in a complex needed for autophagy	Increased CDS
ADAM9	A Disintegrin And Metalloproteinase Domain 9, involved in cell-cell interactions, cell-matrix interactions	Increased 5’-UTR
CALR	Calreticulin, a major Ca(2+)-binding (storage) protein	Decreased 5’-UTR, CDS unchanged
CDKN2C	Cyclin Dependent Kinase Inhibitor 2C, a member of the INK4 family of cyclin-dependent kinase inhibitors that regulate cyclin-dependent kinases	Increased CDS
CLPTM1	Cleft Lip And Palate Associated Transmembrane Protein 1, a paralog of CLPTM1, has a role in susceptibility to cisplatin	Increased 5’-UTR, CDS unchanged
CREBL2	cAMP Responsive Element Binding Protein Like 2, transcription factor associated with lung cancers	Decreased 5’-UTR, increased CDS
CSRP2	Cysteine And Glycine Rich Protein 2, a member of the CSRP family encoding LIM domain proteins involved in cellular differentiation	Increased CDS
DCAF5	DDB1 And CUL4 Associated Factor 5, a ubiquitin ligase that regulates cell proliferation, survival, DNA repair, and genomic integrity	Increased 5’-UTR and CDS
EEF2	Eukaryotic Translation Elongation Factor 2, GTP-binding translation factor that facilitates movement of tRNA-bound peptides from the ribosome A site to the P site.	Decreased CDS
FASN	Fatty Acid Synthase, catalyzes the synthesis of palmitate from acetyl-CoA and malonyl-CoA. Also an mRNA-binding protein.	Increased 5’-UTR, CDS unchanged
FBLN1	Fibulin 1, a secreted glycoprotein that is incorporated into the extracellular matrix	Decreased 5’-UTR, CDS unchanged
GDA	Guanine Deaminase	Decreased CDS
HINT2	Histidine Triad Nucleotide Binding Protein 2, a member of the superfamily of histidine triad proteins, functions in mitochondrial cell death signaling	Increased 5’-UTR, CDS unchanged
HIST1H2AD	Histone Cluster 1 H2A Family Member D	Increased 5’-UTR
H2AFZ	H2A Histone Family Member Z, regulates epithelial-mesenchymal transition, evidence for a role in repressing lncRNA	Increased 5’-UTR
IFIT1	Interferon Induced Protein With Tetratricopeptide Repeats 1, antiviral protein targeting RNAs with 5′-triphosphate ends	Decreased CDS
IGSF9	Immunoglobulin Superfamily Member 9, molecule involved in cell-cell interaction	Increased CDS
KPNB1	Karyopherin Subunit Beta 1, subunit of the importin alpha complex that binds the nuclear localization signal, functions in importing proteins into the nucleus	Increased 5’-UTR, CDS unchanged
LRRC73	Leucine Rich Repeat Containing 73, no known function	Decreased CDS
MECP2	Methyl-CpG Binding Protein 2, mediates transcriptional silencing by binding methyl CpG in DNA, mutations result in Rett syndrome, neurological	Increased CDS
RACK1	Receptor For Activated C Kinase 1, component of the 40S ribosome subunit, involved in signaling between protein kinase pathways and translation	Decreased 5’-UTR
RPL37A	Ribosomal Protein L37A, constituent of the large ribosome subunit	Decreased 5’-UTR
SERTAD3	SERTA Domain Containing 3, a transcriptional coactivator	Increased 5’-UTR and CDS
TBC1D7	Subunit of the tuberous sclerosis TSC1-TSC2 complex	Increased 5’-UTR and CDS
TP53I3	Tumor Protein P53 Inducible Protein 3, an oxidoreductase that is induced by TP53	Increased CDS
TIAM1	T-cell Lymphoma Invasion And Metastasis 1, encodes a RAC1-specific guanine nucleotide exchange factor	Increased 5’-UTR and CDS
TROVE2	TROVE Domain Family Member 2, binds misfolded ncRNAs including Y RNAs	Increased 5’-UTR
ZCCHC3	Zinc finger protein of unknown function, interacts with a large number of proteins, including ELAV1, LIN28B, PAIP1, RACK1, NANOG	Increased 5’-UTR and CDS
ZNF552	Zinc Finger Protein 552, unknown function	Decreased 5’-UTR, increased CDS

This table lists all of the mRNAs (genes) for which changes in Fhit expression alter ribosome binding of the CDS, ribosome binding of the 5’-UTR, or the ratio of ribosomes bound to the 5’-UTR versus coding region (CDS)

It remains to be determined how Fhit or its loss affects changes in translation. The 5’-UTR is a nexus for post-transcriptional gene regulation [34], and with changes in 5’-UTR ribosome occupancy being the feature most characteristic of the targets identified here. We speculate that sequences or structural elements within this region play a major role in determining specificity for their regulation by Fhit. Fhit does not contain any recognizable RNA binding domains, nor has it been identified as a constituent of the mRNP proteome [35–37]. As a member of the histidine triad family Fhit catalyzes the hydrolysis of nucleoside 5′,5′-triphosphates, including m^7^G caps that are the remnants of 3′-5’ mRNA decay [14]. The H1299 cells used here have low levels of the main scavenger decapping enzyme DcpS, and we hypothesize that nucleoside 5′,5′-triphosphates (including but not necessarily limited to m^7^G caps) accumulate in Fhit-negative cells to a level sufficient to compete with eIF4E binding to the capped ends of a limited number of mRNAs. As noted above, sequences or structural elements within the 5’-UTR likely determine susceptibility to this disruption. Indirect targeting of gene expression by the intracellular level of nucleoside 5′,5′-triphosphates is consistent with the observation that Fhit/H96N, which binds nucleoside 5′,5′-triphosphates with high affinity, is nearly as effective as wild-type Fhit in suppressing tumor formation [38, 39].

## Conclusions

In summary, results presented here show that *FHIT* loss is associated with changes in ribosome occupancy of the 5’-UTR and/or coding region of 30 different mRNAs, many of which are associated with cancer. This is consistent with recent findings showing changes in 5’-UTR occupancy of a number of cancer-associated genes precedes appearance of a detectable malignancy. The protein products of Fhit-regulated mRNAs function in a number of different pathways, the diversity of which may help explain the challenges encountered in identifying how *FHIT* loss leads to genome instability and cancer.

## Additional files

Additional file 1: Detailed presentation of methods used for ribosome profiling and bioinformatics analysis. (PDF 84 kb)

Additional file 2: A list of oligonucleotides used. (XLSX 11 kb)

Additional file 3: Scatterplots of duplicate RNA-Seq libraries from Fhit-deficient (E1) and Fhit-expressing (D1) H1299 cells. (PDF 688 kb)

Additional file 4: Excel file listing all of the transcripts identified by RNA-Seq as having increased or decreased steady-state as a function of Fhit expression. These are ordered by fold change and statistical significance. (XLSX 110 kb)

Additional file 5: Scatterplots of duplicate ribosome profiling libraries from Fhit-deficient (E1) and Fhit-expressing (D1) H1299 cells. (PDF 639 kb)

Additional file 6: Metagene analysis showing the 3 nucleotide periodicity of bound ribosomes. (PDF 333 kb)

Additional file 7: Excel file listing all of the transcripts identified by RIBO-Seq as having increased or decreased ribosome occupancy as a function of Fhit expression. These are ordered by fold change and statistical significance. (XLSX 25 kb)

Additional file 8: Scatterplots of average ribosome density of duplicate Fhit- negative (E1) and Fhit-expressing (D1) H1299 cells. (PDF 770 kb)

Additional file 9: Upstream open reading frames within the 5’-UTRs of Fhit-regulated mRNAs. (XLSX 27 kb)

Additional file 10: Transcripts with Fhit-mediated changes in the relative occupancy of 5’-UTR/coding sequence. (XLSX 1582 kb)

## Abbreviations

ARD: Average ribosome density
EMT: Epithelial-mesenchyal transition
uORF: Upstream open reading frame
UTR: Untranslated region
