# Supplementary material for: Impact of FHIT loss on the translation of cancer-associated mRNAs
Source: Mol Cancer. 2017 Dec 28;16:179. doi: 10.1186/s12943-017-0749-x (PMC5745650; doi:10.1186/s12943-017-0749-x)
Supplement: Supplementary file 1 — Detailed presentation of methods used for ribosome profiling and bioinformatics analysis. (PDF 84 kb) [file 12943_2017_749_MOESM1_ESM.pdf]

Additional File 1: Supplemental Materials and Methods

Ribosome profiling and data analysis

Ribosome Profiling

Cells were seeded, cultured, and induced as indicated in Materials and Methods. Cycloheximide (CHX, 100 µg/ml) was added to the medium for 10 min at 37°C. Cells were then washed twice with PBS+CHX (100 µg/ml), scraped, and pelleted by centrifugation at 2500 xg for 5 min at 4°C. Ribosome profiling was performed essentially as outlined in [1] with key alterations detailed below. Cell pellets were lysed in 5 pellet volumes of ice cold polysome lysis buffer (20 mM Tris pH 7.5, 150 mM NaCl, 5 mM $MgCl_2$, 1 mM DTT, 150 µg/ml CHX, 1% Triton X-100, and 0.1 U/µl DNAse 1), incubated on ice for 10 mins with mild agitation every 2 minutes. Cellular debris was pelleted by centrifugation (16,100 xg) for 10 mins at 4°C. 10% of the supernatant was reserved and RNA was harvested as above for sample-matched total cytoplasmic RNA-seq analyses. The remaining material was digested with RNase I (Ambion, 2.5 U/µl) for 45 minutes at 25°C. Superasin (Life technologies, 5 µl per 100 µl of lysate) was added to stop the digestion, samples were vortexed and placed on ice.

Digested samples were loaded into thick-walled ultracentrifuge tubes and a 1 ml sucrose cushion was underlayed. Samples were centrifuged in a Sorvall Discovery M120 SE ultracentrifuge using a S-55A rotor at 54,000 RPM for 4 hours at 4°C. Supernatants were discarded and pelleted RNA was harvested as described in [1], but samples were sequentially eluted off the miRNeasy column using 40 µl and 35 µl of water. At this point, the two elutions were pooled and two additional ribosomal RNA removal steps were added to decrease ribosomal RNA contamination thereby increasing the proportion of ribosome protected fragments (RPFs) in the final libraries. First, as inspired by [2], we generated a pool of biotinylated oligonucleotides (see Additional file 2 for sequences) to hybridize directly to and deplete rRNA sequences that were often incorporated into libraries. The amount of each biotinylated oligo in the pool (17 µl per sample) is dependent upon the prevalence of the contaminating rRNA sequence in previous ribosome profiling library preparations. The proportions of each rRNA depletion oligo are as follows: rRNA_Dep1: (6 µl at 10 µM), rRNA_Dep2: (6 µl at 10 µM), rRNA_Dep3: (3 µl at 10 µM), rRNA_Dep4: (1 µl at 10 µM), rRNA_Dep5 (1 µl at 10 µM). 10 µl of 20x SSC and 17 µl of the premixed depletion pool was added to the RPFs harvested earlier. RNA secondary structures were denatured by incubation at 80°C for 1 min in a thermocycler. Samples were then gradually cooled (-1°C every 20 sec) to 70°C and held at 70°C for 1 minute. Samples were then gradually cooled (-1°C every 30 sec) to 37°C.

After cooling, pre-equilibrated MyOne Streptavidin C1 para-magnetic beads (100 µl of slurry per sample, Life Technologies) were added to each sample and incubated with mixing (350 RPM) in a thermomixer at 37°C for 15 minutes. Tubes were then immediately transferred to a magnetic stand for ~5 minutes and the supernatant (~200 µl) was transferred to a fresh tube. Each sample was split into two tubes and precipitated with the addition of 284 µl H_2O, 4 µl of Glycoblue, and 970 µl of 100% EtOH followed by incubation at -20°C overnight. Remaining RPFs were pelleted by a 35 min centrifugation at 16,100 xg at 4°C. Pellets were rinsed with 70% EtOH and recovered RPFs were resuspended in 28 µl of Tris pH 8.0 and further purified using Ribo-Zero Gold according to the manufacturer's (Illumina) instructions for 1 µg of input RNA. Importantly, the final 50°C incubation step was omitted from the Ribo-Zero Gold protocol and the purified RNAs were resuspended in 5 µl of Tris pH 8.0 . The remainder of the protocol was performed as described in [1] except different biotinylated oligonucleotides were used for subtractive hybridization of circularized ribosome-specific cDNAs and new barcode oligos were

substituted for those listed in [1]. The sequences of the substituted depletion and barcode oligos (cDNA_Dep1 through cDNA_Dep12, and Barcodes 1-4) are listed in Additional file 2.

RNA-Seq libraries and analysis

RNA-Seq was performed by the Genomic Services Lab, HudsonAlpha Institute of Biotechnology, on cytoplasmic RNA harvested from reserved input material from sample-matched H1299 D1 and E1 cultures that were used for ribosome profiling. Libraries were prepared using their automated pipeline and 50 base paired end reads were obtained with an Illumina Hi-Seq 4000. The raw data (FASTQ files) are available online under BioProject accession number PRJNA390535. The two RNA-Seq replicates of D1 cell data initially contained 84,883,380 and 81,745,526 read pairs, respectively, while the two E1 control cell replicate files included 83,998,806 and 63,296,495 read pairs. Sequencing of the RIBO-Seq samples was performed using a HiSeq2500 in the Ohio State Comprehensive Cancer Center Genomics Shared Resource in 50 base pair single-end mode. The two RIBO-Seq replicates of D1 cell data initially contained 68,761,204 and 47,906,118 single-end reads, respectively, while the two E1 cell replicates contained 40,835,106 and 50,341,628 reads, respectively. 3' adapter sequences were trimmed using Skewer [3] and sequence alignment was completed using the STAR aligner (version 2.4.0j) [4], which aligned reads to the hg19 genome. Alignments were then converted to BAM format using SAMtools [5]. Gene expression was measured by counting aligned reads that overlapped with Refseq gene annotations using HTSeq [6], which preprocesses RNA-seq and ribosome profiling data for differential expression analysis. Un-normalized read counts were then used to detect differential expression with the DESeq2 analysis toolset (version 1.0.17) [7] by calculating the geometric mean across all samples for each gene. Counts for each gene are divided by this mean correcting for library size. DESeq2 then fits negative binomial generalized linear models for each gene and tests for significance using the Wald statistical test. Additionally, unnormalized read counts were used with RiboDiff [8] to detect genes with changes in translation across experimental conditions. The application fits generalized linear models to estimate the over-dispersion of RNA-seq and ribosome profiling measurements separately, and uses mRNA abundance and ribosome occupancy to perform a statistical test for differential translation.

To better understand how Fhit's presence affects all genes, we focused on both a gene's 5' UTR and its coding region. This allowed us to localize any statistically significant effects within either region that might have otherwise been overlooked. In order to mitigate influence either region may have on the other, we removed, *in silico*, the first 25 bases downstream from the first base in the annotated start codon in the coding regions. Then using HTseq and RiboDiff, we measured the differential translation of the 5' UTR and coding regions separately. 18,283 single Refseq mRNA isoforms for each gene were chosen from the HUGO Gene Nomenclature Committee database [9] using data retrieved in March 2017, and annotations were obtained from the USCS Table Browser [10], also in March 2017.

Total alignment rates for the Fhit-positive RNA-seq replicates were 57% (13% multi-mapped) and 48% (11% multi-mapped), with 21% and 25% aligning to regions of r/tRNA. Similarly, for the Fhit-negative RNA-seq replicates 44% (9% multi-mapped) and 54% (12% multi-mapped) of the reads were aligned with 27% and 24% aligning to regions of r/tRNA. Total alignment rates for the Fhit-positive RIBO-Seq replicates were 16% (5% multi-mapped) and 16% (6% multi-mapped), with 77% and 78% aligning to regions of r/tRNA. Similarly, for the Fhit-negative RIBO-Seq replicates 9% (3% multi-mapped) and 12% (6% multi-mapped) of the reads were aligned, with 79% and 81% aligning to regions of r/tRNA.

Using the two different regions of the 18,283 annotated genes, we measured differential expression of both the RNA-seq and RIBO-Seq data. For the shortened coding regions 12,416

genes (RNA-seq) and 7,735 genes (RIBO-Seq) were tested for statistical significance. In total 854 and 91 genes, respectively, were found to be differentially expressed to some degree based on a multiple-testing adjusted p-value < 0.05. For the 5' UTR 7,619 genes (RNA-seq) and 11,448 genes (RIBO-Seq) were tested. It was found that only one gene (RPL37A, NM_000998) from the RIBO-Seq data had a multiple-testing adjusted p-value < 0.05 while the RNA-seq contained 60 genes whose 5' UTRs were found to be differentially expressed with multiple-testing adjusted p-value < 0.05.

As described above, raw count data from both regions considered for each of the 18, 283 genes for both RNA-seq and RIBO-Seq were then processed with RiboDiff to test for changes in translational efficiency. For the shortened coding regions the change in translational efficiency of 10 genes were found to be statistically significant (adjusted p < 0.05) and are highlighted in red in Figure 1E. Only seven genes were found to have a statistically significant change in translational efficiency when only count data from the 5' UTR was measured, as shown in Figure 3.

From several of the gene coverage graphs, it appears that additional translation events could be occurring within the 5' UTR, presumably at uORFs. Similar to [11] translation of the 5' UTR serves well as a proxy for uORF translation. Thus we calculated the relative 5' UTR translation as the ratio of Ribo-Seq counts in a gene's 5' UTR to its Ribo-Seq counts within the shortened CDS region. The change in translational efficiency for the relative 5' UTRs of all genes was then calculated using RiboDiff. For this targeted RiboDiff analysis, the Ribo-Seq reads in the CDS replaced RNA-seq data and compared to the Ribo-Seq reads in the 5' UTR. This method identified 19 genes having statistically significant (p< 0.05) changes in the translational efficiency within their 5' UTRs.

References

1. Ingolia NT, Brar GA, Rouskin S, McGeachy AM, Weissman JS. The ribosome profiling strategy for monitoring translation in vivo by deep sequencing of ribosome-protected mRNA fragments. Nat Protoc. 2012;7:1534-1550.
2. Subtelny AO, Eichhorn SW, Chen GR, Sive H, Bartel DP. Poly(A)-tail profiling reveals an embryonic switch in translational control. Nature. 2014;508:66-71.
3. Jiang H, Lei R, Ding SW, Zhu S. Skewer: a fast and accurate adapter trimmer for next-generation sequencing paired-end reads. BMC Bioinformatics. 2014;15:182.
4. Dobin A, Davis CA, Schlesinger F et al. STAR: ultrafast universal RNA-seq aligner. Bioinformatics. 2013;29:15-21.
5. Li H, Handsaker B, Wysoker A et al. The Sequence Alignment/Map format and SAMtools. Bioinformatics. 2009;25:2078-2079.
6. Anders S, Pyl PT, Huber W. HTSeq--a Python framework to work with high-throughput sequencing data. Bioinformatics. 2015;31:166-169.
7. Anders S, Huber W. Differential expression analysis for sequence count data. Genome Biol. 2010;11:R106.
8. Zhong Y, Karaletsos T, Drewe P et al. RiboDiff: detecting changes of mRNA translation efficiency from ribosome footprints. Bioinformatics. 2017;33:139-141.
9. Gray KA, Yates B, Seal RL, Wright MW, Bruford EA. Genenames.org: the HGNC resources in 2015. Nucleic Acids Res. 2015;43:D1079-85.
10. Karolchik D, Hinrichs AS, Furey TS et al. The UCSC Table Browser data retrieval tool. Nucleic Acids Res. 2004;32:D493-6.
11. Sendoel A, Dunn JG, Rodriguez EH et al. Translation from unconventional 5' start sites drives tumour initiation. Nature. 2017;541:494-499.
