# Supplementary material for: Impact of FHIT loss on the translation of cancer-associated mRNAs
Source: Mol Cancer. 2017 Dec 28;16:179. doi: 10.1186/s12943-017-0749-x (PMC5745650; doi:10.1186/s12943-017-0749-x)
Supplement: Supplementary file 3 — Scatterplots of duplicate RNA-Seq libraries from Fhit-deficient (E1) and Fhit-expressing (D1) H1299 cells. (PDF 688 kb) [file 12943_2017_749_MOESM3_ESM.pdf]

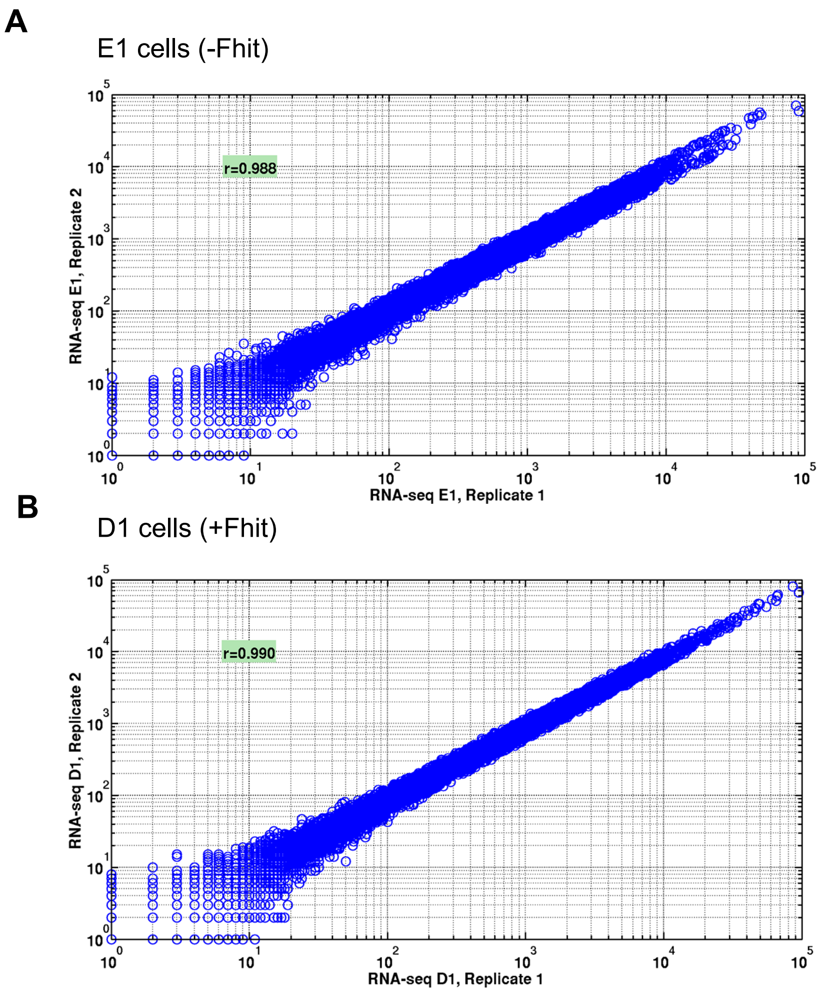

Additional file 3

Comparison of RNA-Seq libraries from Fhit-negative (E1) and Fhit-positive (D1) H1299 cell lines. Shown are scatterplots of duplicate RNA-Seq libraries from Ponasterone A-treated H1299 cells with the Spearman coefficient for each shown in the green box. The E1 cell line is stably transfected with empty vector and the D1 cell line carries an inducible Fhit transgene.
