# Supplementary material for: Impact of FHIT loss on the translation of cancer-associated mRNAs
Source: Mol Cancer. 2017 Dec 28;16:179. doi: 10.1186/s12943-017-0749-x (PMC5745650; doi:10.1186/s12943-017-0749-x)
Supplement: Supplementary file 5 — Scatterplots of duplicate ribosome profiling libraries from Fhit-deficient (E1) and Fhit-expressing (D1) H1299 cells. (PDF 639 kb) [file 12943_2017_749_MOESM5_ESM.pdf]

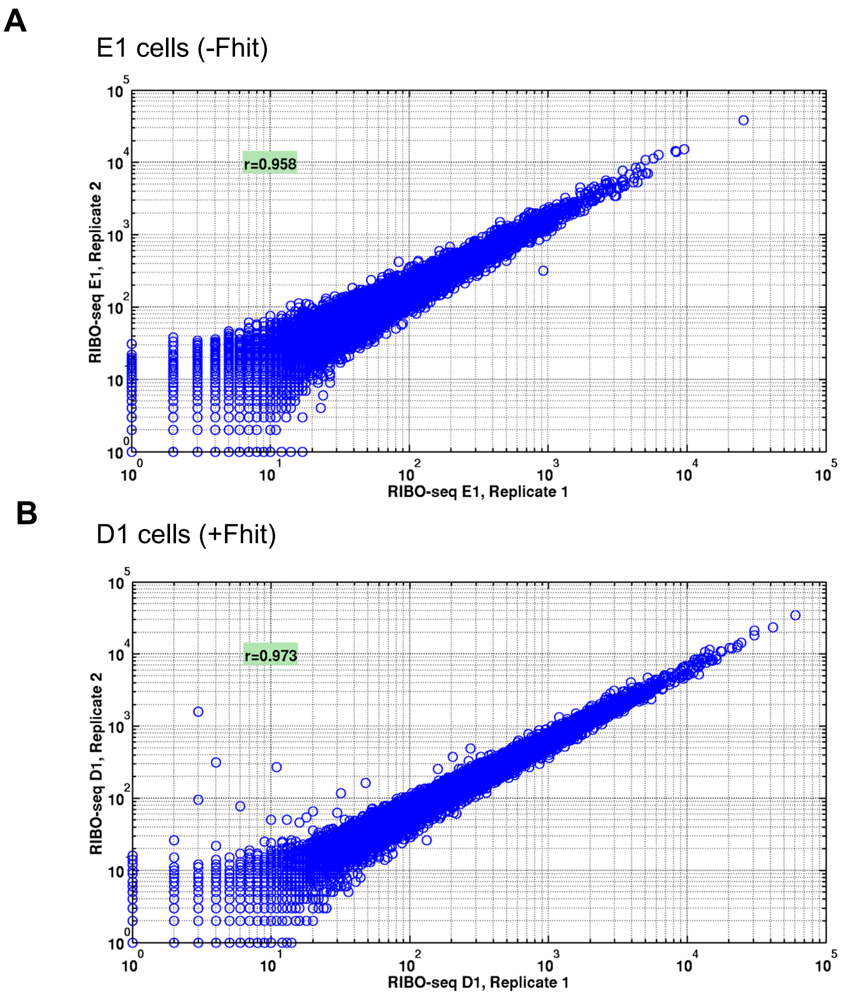


## Additional file 5

**Comparison of ribosome profiling libraries from Fhit-deficient (E1) and Fhit-expressing (D1) H1299 cells.** Shown are scatterplots of duplicate ribosome profiling libraries from Ponasterone A-treated H1299 cells. The Spearman coefficient for each is shown in the green box. The E1 cell line is stably transfected with empty vector and the D1 cell line carries an inducible Fhit transgene.
