# Supplementary material for: Impact of FHIT loss on the translation of cancer-associated mRNAs
Source: Mol Cancer. 2017 Dec 28;16:179. doi: 10.1186/s12943-017-0749-x (PMC5745650; doi:10.1186/s12943-017-0749-x)
Supplement: Supplementary file 6 — Metagene analysis showing the 3 nucleotide periodicity of bound ribosomes. (PDF 333 kb) [file 12943_2017_749_MOESM6_ESM.pdf]

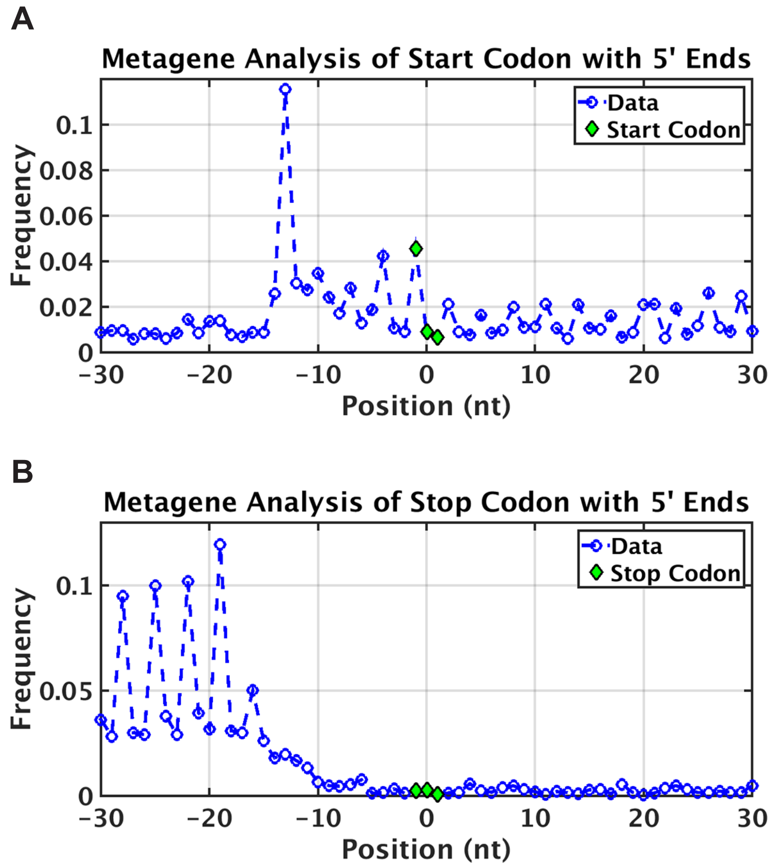

Additional file 6

Metagene analysis shows periodicity of bound ribosomes. The 5' ends of reads of each of the ribosome protected fragments were mapped with respect to start (**A**) and stop (**B**) codons. This confirmed a 3 nucleotide periodicity consistent with the triplicate nature of codons and the peak 13 nucleotides upstream of start codons is consistent with ribosomes bound at these locations.
