# Supplementary material for: Impact of FHIT loss on the translation of cancer-associated mRNAs
Source: Mol Cancer. 2017 Dec 28;16:179. doi: 10.1186/s12943-017-0749-x (PMC5745650; doi:10.1186/s12943-017-0749-x)
Supplement: Supplementary file 8 — Scatterplots of average ribosome density of duplicate Fhit- negative (E1) and Fhit-expressing (D1) H1299 cells. (PDF 770 kb) [file 12943_2017_749_MOESM8_ESM.pdf]

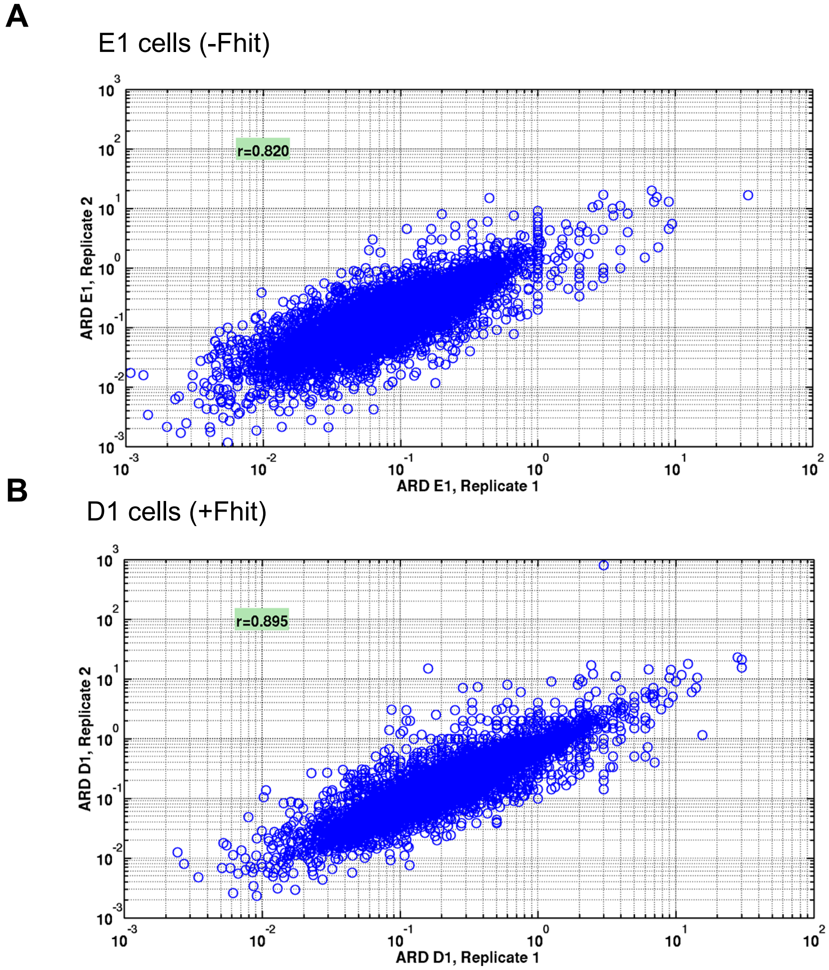

Additional file 8

Average ribosome density of Fhit- negative and Fhit-expressing H1299 cells. Shown are scatterplots of average ribosome densities from ribosome profiling libraries of H1299 cells. The Spearman coefficient for each plot is shown in the green box. The E1 cell line (**A**) is stably transfected with empty vector and the D1 cell line (**B**) carries an inducible Fhit transgene. Both cell lines were treated with Ponasterone A.
